# Hubei's Core Response Policies in the Early Stage of COVID-19

**DOI:** 10.1155/2021/6610045

**Published:** 2021-05-28

**Authors:** Yuyao Zhang, Leiyu Shi, Haiqian Chen, Xiaohan Wang, Gang Sun

**Affiliations:** ^1^Department of Health Management, School of Health Management, Southern Medical University, Guangzhou, Guangdong 510515, China; ^2^Department of Health Policy and Management, Bloomberg School of Public Health, Johns Hopkins University, Baltimore, MD 21205, USA

## Abstract

**Background:**

This study is aimed at confirming the effectiveness of nonpharmaceutical interventions during the COVID-19 outbreak in Hubei, China.

**Methods:**

The data are all from the epidemic information released by the National Health Commission of the People's Republic of China and the Health Commission of Hubei Province. We used the multivariable linear regression by the SPSS 19.0 software: the cumulative number of confirmed cases, the cumulative number of cured cases, and the number of daily severe cases were taken as dependent variables, and the six policies, including the Joint Prevention and Control Mechanism of the State Council, lockdown Wuhan city, the first-level response to public health emergencies, the expansion of medical insurance coverage to suspected patients, mobile cabin hospitals, and counterpart assistance in Hubei province, were gradually entered into multiple linear regression models as independent variables.

**Results:**

The factors influencing the cumulative number of diagnosed cases ranged from large to small: mobile cabin hospitals and the expansion of medical insurance coverage to suspected patients. The factors influencing the cumulative number of cured cases ranged from large to small: counterpart support medical teams in Hubei province and mobile cabin hospitals. The factors influencing the number of daily severe cases ranged from large to small: mobile cabin hospitals and the expansion of medical insurance coverage to suspected patients.

**Conclusion:**

The mobile cabin hospital is a major reason for the successfully defeating COVID-19 in China. As COVID-19 pandemic spreads globally, the mobile cabin hospital is a successful experience in formulating policies to defeat COVID-19 for other countries in the outbreak phase.

## 1. Introduction

COVID-19 continues to spread around the world. According to WHO, as of 31 March 2021, there were 128,540,982 confirmed cases and 2,808,308 deaths worldwide [1]. The COVID-19 epidemic prevention and control is a tough test to the governance system and governance ability of all countries [2–4]. As the worst-hit province in China, Hubei government resolutely adopted a series of prevention and control policies during the outbreak period in view of the large number of infected people, the rapid spread of the virus, and the shortage of health resources, which played a key role in defeating COVID-19 in Hubei province and even the whole of China.

In order to contain the ongoing outbreak of COVID-19 in Hubei province, from January 20 to February 12, the Hubei government has adopted a series of prevention and control policies, which are summarized in the following six items (Table 1): Hubei Province New Coronavirus Infection Pneumonia Prevention and Control Headquarters, lockdown Wuhan city, first-level response to public health emergencies, the expansion of medical insurance coverage to suspected patients, mobile cabin hospitals, and the counterpart assistance in Hubei province (excluding Wuhan). We used multivariable linear regression models to analyze the association between nonpharmaceutical policies and the number of cases in order to provide policy experience on global COVID-19 pandemic.

## 2. Data and Methods

### 2.1. Data

The data are all from the epidemic information released by the National Health Commission of the People's Republic of China and the Health Commission of Hubei Province [5, 6]. We collated and integrated the data. The research indicators included the cumulative number of confirmed cases and the cumulative number of cured cases.

### 2.2. Methods

The data were sorted out by Excel, and the effects of prevention and control policies were studied based on the Hubei's data. Graphs and tables were combined, and descriptive analysis was combined with statistical inference.

Graphs and tables were performed to show the trend of cumulative confirmed cases and the trend of cumulative cured cases in Hubei province from January 10, 2020, to March 10, 2020. We used the multivariable linear regression by the SPSS 19.0 software: the cumulative number of confirmed cases and the cumulative number of cured cases were taken as dependent variables, and the six policies adopted by Hubei government were gradually entered into multiple linear regression models as independent variables.

## 3. Results

### 3.1. Hubei's COVID-19 Epidemic Trends in the Outbreak Phase

On January 10, 2020, the National Health Commission of the People's Republic of China began to release COVID-19 epidemic. As of 24 : 00 on March 10, 2020, Figure 1 showed the trend of cumulative confirmed cases and the trend of cumulative cured cases in Hubei, China.

From January 10 to February 18, 2020, the cumulative number of confirmed cases showed a trend of rapid growth, and the cumulative number of cured cases increased slowly. After February 18, the growth trend of the cumulative confirmed cases gradually slowed down, and the cumulative number of cured cases increased significantly. As of March 10, the cumulative number of confirmed cases was close to the cumulative number of cured cases. Therefore, January 10 to February 18 can be regarded as the outbreak period of COVID-19 in Hubei province. This study analyzed the data in this period.

### 3.2. Factors Affecting Cumulative Confirmed Cases in Hubei Province

The multivariable linear regression model was adopted to take the cumulative number of confirmed cases as the dependent variable *Y* and the six major prevention and control policies ①~⑥ (Table 1) as the independent variable *X*_1_ ~ *X*_6_. Through stepwise regression analysis, independent variables entering into the model are *X*_5_, *X*_4_, *P* ≤ 0.001, and *P* ≤ 0.001 (Table 2). It can be seen that the mobile cabin hospitals and the expansion of medical insurance coverage to suspected patients have significant impact on the cumulative confirmed cases.

According to Table 2, the regression model can be written as *Y* = −55163.462 + 28877.857*X*_5_ + 7926.144*X*_4_. The results of variance analysis showed *F* = 426.825 and *P* ≤ 0.001, indicating that this model was statistically significant. The adjusted *R*^2^ = 0.970 shows that the model fits better. The coefficient of regression equation indicates the influence of independent variable on dependent variable. Based on the magnitude of coefficients, it can be seen that influencing factors of cumulative confirmed cases range from large to small: the mobile cabin hospitals and the expansion of medical insurance coverage to suspected patients.

### 3.3. Factors Affecting Cumulative Cured Cases in Hubei Province

The multivariable linear regression model was adopted to take the cumulative number of cured cases as the dependent variable *Y* and the six major prevention and control policies ①~⑥ (Table 1) as the independent variable *X*_1_ ~ *X*_6_. Through stepwise regression analysis, independent variables entering into the model are *X*_6_, *X*_5_, *P* ≤ 0.001, and *P* = 0.004 (Table 3). It can be seen that the counterpart support medical teams in Hubei province (excluding Wuhan) and the mobile cabin hospitals have significant impact on the cumulative cured cases.

According to Table 3, the regression model can be written as *Y* = −5806.000 + 4458.143*X*_6_ + 1435.357*X*_5_. The results of variance analysis showed *F* = 124.659 and *P* ≤ 0.001, indicating that this model was statistically significant. The adjusted *R*^2^ = 0.864 shows that the model fits better. The coefficient of regression equation indicates the influence of independent variable on dependent variable. Based on the magnitude of coefficients, it can be seen that influencing factors of cumulative cured cases range from large to small: the counterpart support medical teams in Hubei province (excluding Wuhan) and the mobile cabin hospitals.

## 4. Discussion

The sudden outbreak of the epidemic put an overwhelming burden on medical resources and led to treatment delays. The unchecked virus triggered a high mortality rate, posing the biggest challenge in the early fight against the epidemic. In the absence of vaccines or treatment protocols, self-isolation, as a standard quarantine measure, proved to be the most effective nonmedical means to stop the spread of the virus [7]. The arrival of counterpart medical team greatly improved the ability to receive and treat COVID-19 patients in Hubei province. Expanding health insurance coverage to suspected patients addressed cost concerns for COVID-19 patients, especially suspected patients. Mobile cabin hospitals reduced the chances of cross infection in communities to a large extent. This is consistent with the results of this study. The factors defeating the COVID-19 in outbreak phase in Hubei province are mobile cabin hospitals, counterpart support medical teams, and the expansion of medical insurance coverage to suspected patients.

This study found that during the outbreak period of COVID-19, mobile cabin hospitals had a significant impact on the cumulative confirmed cases and the cumulative cured cases. In addition, mobile cabin hospitals ranked first among the factors influencing the cumulative confirmed cases, which further demonstrated the pivotal role of mobile cabin hospitals in defeating COVID-19 in Hubei province. Mobile cabin hospital is a field mobile medical platform that can be swiftly transported and built in the form of a medical mobile cabin with an integration of medical services and medical technology support [8]. Mobile cabin hospitals have the same treatment level as hospitals of class II grade A, which will improve the treatment efficiency of centralized admission [9]. The first mobile cabin hospital was constructed on February 3, 2020, and was put in official operation on February 5. On February 27, the situation of “a hospital bed being hard to come by” changed fundamentally as the quantity of hospital beds outstripped patient demand in 20 days [10]. The 16 mobile cabin hospitals in Wuhan admitted up to 12,000 patients with mild symptoms, accounting for more than a quarter of the infected patients [7]. On average, one of every four confirmed patients received treatment in the mobile cabin hospital [11].

The study also found that the expansion of medical insurance coverage played an important role. Roughly estimated, a highly suspected patient will have to pay around RMB 5,000, some patients with common symptoms may have to pay RMB 10,000, and critical patients may even have to pay up to RMB 1 million or more [12]. The expansion of medical insurance coverage to suspected patients [13] can not only ensure that the confirmed patients receive free treatment, but also exempt the individual burden of the suspected patients. It greatly promotes the patients with mild and suspected diseases to accept isolation observation consciously, so as to avoid cross infection in a wider range. The mobile cabin hospitals have provided sufficient isolation beds and professional nursing services for the large number of suspected and mild patients, which has earned valuable time for the control with COVID-19.

The study found that counterpart assistance was effective as well [14]. The terrible thing about COVID-19 is that the highly contagious nature has led to an influx of patients in a short period of time, resulting in a severe shortage of health resources, especially medical staff. And the health system faces collapse at any moment. China offers a solution by taking the nationwide “pairing assistance” measure, mobilizing 29 provinces to alleviate pressure on cities in Hubei province [15]. On February 12, 2020, the counterpart support teams in Hubei province are in place, which can quickly alleviate the conflict between excessive suspected and confirmed cases and insufficient medical personnel, and improve the medical treatment capacity of all cities (except Wuhan) in Hubei province. It shows that the counterpart medical team has a significant impact on the cumulative number of cured cases, which is consistent with the conclusion of this study. A total of 94 medical teams and more than 8,000 healthcare workers served in mobile cabin hospitals [16]. It is very important for the mobile cabin hospitals with a large number of mild patients.

Mobile cabin hospitals inserted an additional level of care into the Chinese health system and thus served a strategic triage function for patients with COVID-19 [17]. Patients with mild to moderate COVID-19 who met additional admission criteria [14, 18] are admitted to mobile cabin hospitals for supportive treatment, the severe patients are transferred to specialized hospitals for further treatment, and the critical patients are placed in the ICU of designated hospitals for life-supporting treatment such as ECMO and endotracheal intubation [19, 20]. The operation of mobile cabin hospitals can quickly cut off the source of infection and the way of spread to achieve “admit and treat everyone necessary,” so that the patients who should receive the most active treatment get high-quality medical resources.

There are two innovation points in our study as follows. On the one hand, we adopted multivariate linear regression models to analyze the effects of nonpharmacological interventions during the COVID-19 outbreak phase in Hubei, China. On the other hand, we found that the mobile cabin hospital was a common factor influencing the cumulative number of confirmed cases and the cumulative number of cured cases. We consider this to be an important finding in the quantitative study of COVID-19.

## 5. Conclusions

The mobile cabin hospital is a critical way for the successfully defeating COVID-19 in Hubei, China. In the outbreak phase of COVID-19, the mobile cabin hospitals had a critical impact on the cumulative confirmed cases and the cumulative cured cases; the expansion of medical insurance coverage to suspected patients addressed the financial burden of both confirmed and suspected patients; the counterpart support medical teams ensured the normal operation of mobile cabin hospitals. As the COVID-19 pandemic spreads globally, the study provides successful policy experience to defeat the COVID-19 for other countries.

## Figures and Tables

**Figure 1:**
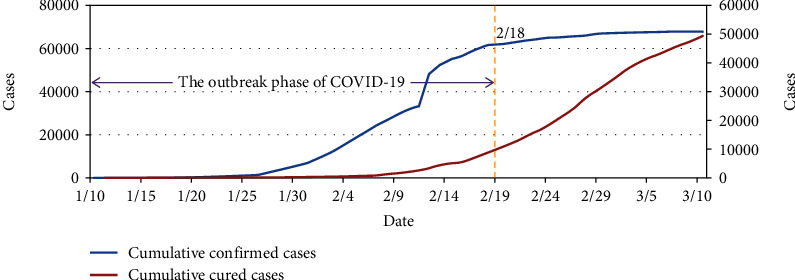
COVID-19 epidemic trends in Hubei province.

**Table 1:** Six major prevention and control policies.

No.	Date	Policies	Effect
①	2020.1.22	Establishing the Hubei Province New Coronavirus Infection Pneumonia Prevention and Control Headquarters	The headquarters will coordinate COVID-19 prevention and control in Hubei province.
②	2020.1.23	Locking down Wuhan city	Transregional spread of the COVID-19 epidemic will be contained.
③	2020.1.24	Initiating the first-level (highest level) response to public health emergencies	Hubei province will enter a highly alert state.
④	2020.1.27	Expanding the coverage of the previous comprehensive guarantee policy for diagnosed patients to suspected patients who meet the diagnosis and treatment guidelines of National Health Commission	Patients will not be delayed because of the cost.
⑤	2020.2.5	Launching the first mobile cabin hospital (Jianghan mobile cabin hospital)	The pressure of hospital admission will be greatly relieved in the short term.
⑥	2020.2.12	The counterpart support medical teams in Hubei province (excluding Wuhan) in place	Through “One province Supports One City,” the entire country will support the epidemic prevention and control in Hubei province.

**Table 2:** Association between cumulative confirmed cases and nonpharmaceutical policies.

Model	Unstandardized coefficients		Standardized coefficients	*t*	Sig.	Collinearity statistics	
	*B*	Std. error	Beta			Tolerance	VIF
(Constant)	-55163.462	2216.888		-24.883	.000		
Mobile cabin hospitals	28877.857	1926.979	.531	14.986	.000	.606	1.650
The expansion of medical insurance coverage to suspected patients	7926.144	1486.113	.189	5.333	.000	.602	1.661

Note: *R*^2^ = 0.970, *F* = 426.825, and *P* ≤ 0.001.

**Table 3:** Association between cumulative cured cases and nonpharmaceutical policies.

Model	Unstandardized coefficients		Standardized coefficients	*t*	Sig.	Collinearity statistics	
	*B*	Std. error	Beta			Tolerance	VIF
(Constant)	-5806.000	477.707		-12.154	.000		
The counterpart support medical teams in Hubei province (excluding Wuhan)	4458.143	468.777	.722	9.510	.000	.606	1.650
Mobile cabin hospitals	1435.357	373.441	.292	3.844	.004	.606	1.650

Note: *R*^2^ = 0.864, *F* = 124.659, and *P* ≤ 0.001.

## Data Availability

The data are all from the epidemic information released by the National Health Commission of the People's Republic of China and the Health Commission of Hubei Province (http://www.nhc.gov.cn/xcs/xxgzbd/gzbd_index.shtmlhttp://wjw.hubei.gov.cn/bmdt/ztzl/fkxxgzbdgrfyyq/xxfb/).
